# Individualism Increases the Influence of Perceived Competence of Older Adults on Attitudes Toward Them

**DOI:** 10.1093/geroni/igaa057.1043

**Published:** 2020-12-16

**Authors:** Amber Xuqian Chen, Helene Fung

**Affiliations:** 1 The Chinese University of Hong Kong, Hong Kong, China; 2 The Chinese University of Hong Kong, Sha Tin, China

## Abstract

Negative views of ageing can lower respect for older adults.Yet, negative views of ageing vary across cultures. Asian collectivistic cultures are assumed to respect older adults more than Western individualistic cultures do. However, recent empirical findings on this cross-cultural comparison have suggested that negative attitudes toward older people are also prevalent, or even more evident in collectivistic cultures than individualistic cultures. Using data from the sixth wave of the World Values Survey, a dataset consisting of 75,650 individuals from 56 societies, we employed Linear Mixed Modeling to test the association between perceived competence of older adults and respect towards them. We also explored and the moderating role of culture on this association. In the present study, perceived competence of older adults was indexed as a proportional score representing the relative perception of competence (i.e. relative competence perception = competence / (competence + friendliness). Results showed that individuals tended to respect older adults who were more competent or friendly. Furthermore, individuals who were more individualistic respected older adults more when older adults were perceived to be more competent relative to friendly. This pattern was reversed in individuals who were less individualistic. These findings suggest that whether people who differ on personal individualistic values respect older adults depends on whether older adults are perceived to be competent versus friendly. Findings from this study highlight the importance of changing cultural values on ageism attitudes, especially the potential effects of rising individualism on negative attitudes of ageing in Asia.

